# Technology-enabled hybrid cardiac rehabilitation: Qualitative study of healthcare professional and patient perspectives at three cardiac rehabilitation centres in England

**DOI:** 10.1371/journal.pone.0319619

**Published:** 2025-03-07

**Authors:** Sarah Damery, Janet Jones, Alexander Harrison, Sebastian Hinde, Kate Jolly

**Affiliations:** 1 Department of Applied Health Sciences, University of Birmingham, Birmingham, United Kingdom,; 2 National Audit of Cardiac Rehabilitation, Department of Health Sciences, University of York, York, United Kingdom,; 3 Centre for Health Economics, University of York, York, United Kingdom; University of Oxford Nuffield Department of Clinical Medicine: University of Oxford Nuffield Department of Medicine, UNITED KINGDOM OF GREAT BRITAIN AND NORTHERN IRELAND

## Abstract

Coronary heart disease (CHD) is a leading cause of death in the UK. Clinical guidelines recommend cardiac rehabilitation (CR), including health education, cardiovascular risk reduction advice, physical activity and stress management components. However, uptake of standard in-person, group-based CR is only around 50%. Hybrid cardiac rehabilitation (CR), combining in-person and remote service delivery, may improve CR uptake and reduce inequalities in service access. This study used focus groups and semi-structured interviews to explore staff and patient experiences of using the Active^+^me REMOTE hybrid CR app, a cloud-based platform providing access to education modules, behaviour change support, live exercise classes, physical activity and health monitoring across three sites in the East of England. Twelve staff and six patients participated. Topic guides explored participants’ experiences of delivering or receiving hybrid CR, barriers and facilitators associated with the hybrid CR pathway, and implications for future implementation of Active^+^me REMOTE. Qualitative data were collected remotely, audio-recorded and independently transcribed. Staff data were analysed deductively, using the Consolidated Framework for Implementation Research (CFIR). Patient data were analysed inductively using thematic analysis. Despite some technical issues and governance delays, Active^+^me REMOTE was perceived as acceptable, convenient and allowed tailoring of support to meet patients’ needs and circumstances. Data upload from wearable devices (blood pressure monitors) allowed staff to monitor patients’ progress and empowered patients to direct their recovery. Staff initially felt they should screen patients to ensure that hybrid CR was offered to digitally literate, physically active individuals, although screening became less common as staff familiarity with the app increased. Findings suggest that effective implementation of hybrid CR requires system-level resource to facilitate governance approvals and embed hybrid CR delivery as standard care. Sufficient time must be allowed for staff training and to support patient enrolment to hybrid services. The study was registered on 3/7/2023 (ISRCTN320764).

## Introduction

Coronary heart disease (CHD) is one of the leading causes of death in the UK [1], and clinical guidelines recommend cardiac rehabilitation (CR) as a key element of CHD care pathways [2,3]. CR is a complex intervention which includes health education, advice on cardiovascular risk reduction, physical activity and stress management [4]. In the UK, the British Association for Cardiovascular Prevention and Rehabilitation (BACPR) outlines six core CR components: health behaviour change and education; lifestyle risk factor management; psychosocial health; medical risk factor management; long term strategies, and audit and evaluation [5]. The National Institute for Health and Care Excellence (NICE) recommends CR for people with stable heart failure and those recovering from myocardial infarction (MI), specifying that CR should be offered in a choice of venues and times of day, with equal accessibility to people with different demographic characteristics [2,3].

Despite national guidance and the known benefits of CR [6], the UK National Audit of Cardiac Rehabilitation (NACR) recently reported that the average uptake of in-person, group-based CR is around 50% of those deemed eligible, with substantial variation by gender, rural or urban status, ethnicity and socioeconomic deprivation [7,8]. CR classes conducted in hospitals may be difficult to reach due to distance from patients’ homes [9], or travel costs and time required [10–12], and fixed class times are challenging for people in full-time employment, shift workers or those with family or caring responsibilities [13,14]. There may also be embarrassment related to attending group-based CR sessions, and people from some ethnic groups may experience language barriers and find a mixed-sex learning and exercise setting uncomfortable [15].

Before the COVID-19 pandemic, most CR programmes in England were delivered in-person (two classes per week for around 11 weeks) typically combining supervised exercise and education [7]. The pandemic necessitated the use of remote technologies to deliver CR classes, and some CR services have continued offering specific elements of CR programmes remotely, through ‘hybrid’ approaches combining in-person and remote delivery. There is no standard composition of a hybrid CR programme, but hybrid CR typically combines both in-person, centre-based exercise and monitored home-based exercise, usually facilitated through a software app in which at least two of the core components of CR are offered (e.g., education, exercise). Patients have the flexibility to choose their mode of participation and can attend some in-person sessions if they wish, whilst undertaking home-based CR for the remainder of their CR programme. Hybrid CR aims to save time, reduce National Health Service (NHS) costs and staffing pressures, and enable more patients to engage with services. The use of hybrid CR was recommended by NACR in 2021 [7], and also aligns with the objective of the NHS Long Term Plan to increase CR uptake to 85% by 2028 [16]. However, despite the potential for technology to increase CR uptake and adherence, the effectiveness of hybrid programmes in achieving clinical benefits comparable to in-centre programmes has not been definitively established. There is also limited data on patient and staff perspectives about hybrid CR services. As part of a larger study that aimed to assess the clinical and health economic outcomes associated with hybrid CR adoption across multiple NHS sites, we undertook qualitative work in three NHS Trusts to explore the patient and staff experience of using a hybrid model of CR.

## Materials and methods

Methods are reported according to the COnsolidated criteria for REporting Qualitative research (COREQ) guidelines [17]. The COREQ checklist is supplied as supplementary information [S1 Table].

### Study design and setting

The study used mixed methods and included a quantitative analysis of routinely collected audit data from the National Audit of Cardiac Rehabilitation (NACR), a health economic model, and qualitative interviews or focus groups with patients and staff delivering CR at three NHS centres in the East of England who were implementing the Active+me REMOTE hybrid CR model as part of an initiative supported by their respective Integrated Care Boards (ICBs). This paper reports the findings of the qualitative component of the study.

### The Active^+^me REMOTE hybrid cardiac rehabilitation intervention

The Active^+^me REMOTE product (Aseptika Ltd – www.activ8rlives.com) was previously evaluated as a fully remote CR programme in an uncontrolled service evaluation [18]. It is a cloud-based platform with an app for patients to access via Bluetooth-enabled smart phone and secure NHS login. Active^+^me REMOTE was designed using behaviour change techniques [19] and has been certified as a medical device that meets NHS Health and Social Care Digital Technology Assessment Criteria (DTAC). In this study, Active^+^me REMOTE was used as part of a hybrid delivery approach to CR (Table 1). The Active+me REMOTE hybrid CR programme combines specifically tailored, remotely delivered group exercise classes, health coaching, health monitoring via wearable devices provided by Aseptika according to clinical need (e.g., heart rate, blood pressure, physical activity tracking, pulse oximeter, body mass scales) allowing healthcare staff to remotely monitor patient performance through the Activ8rlives platform and patients to review their own health information and progress throughout their 12-week hybrid CR programme. Wearable devices could be linked using Bluetooth to any smart device using Android, Kindle or Apple platforms. During live remote exercise sessions, patients could enter and show privately, their Rated Perceived Exertion values to the instructor, indicating they were achieving an appropriate level of exertion. Patients were able to attend in-person CR at any time if they wished to do so.

**Table 1:** Features of the Active^+^me REMOTE hybrid CR intervention.

Objective	Active^+^me REMOTE feature
Content	Suite of modules for lifestyle education (e.g., weight management), behaviour change support, live exercise classes, physical activity and health monitoring tools
Personalised CR delivery	Patients initially attended CR in person and were supported to engage with remote CR delivery at a pace appropriate to their individual needs
Sites able to choose features	Participating sites were able to choose the app functions that were accessible to their patients: education only, education and exercise (two sites), or education, exercise and food diaries (one site)
Healthcare professional remote monitoring	Healthcare professionals could communicate with patients throughout their 12-week CR programme, monitor patient progress towards achieving goals, and assess engagement with CR using data transmitted from patients’ Bluetooth accessory devices (e.g., blood pressure monitors) in real-time or asynchronously
Patient choice	Patients could attend an in-person CR session at any point if they required in-person support
Availability of technical support	The Active^+^me REMOTE developers were available to patients and CR staff throughout the study to troubleshoot any issues with the app or associated technology

### Participant sampling and recruitment

Data were collected from CR staff and managers to explore their experience of implementing the hybrid Active^+^me REMOTE programme at their site, and adult patients invited to attend hybrid CR at those sites. Any CR staff member or manager at sites delivering hybrid CR using Active+me REMOTE was eligible to participate, as were all patients who had been invited to attend the hybrid CR programme for their heart condition, regardless of whether or not they engaged with the programme. It was anticipated that around 20 staff and 20 patients would be required for thematic saturation.

All CR staff and managers at each site were invited to participate in a focus group or interview by the CR lead nurse or administrator via email. Potential participants responded directly to the qualitative research fellow (JJ) who provided a Participant Information Leaflet (PIL) and consent form. Reminders were sent two weeks after the initial invitation. All staff who wished to participate were invited to attend an online focus group of up to eight people or an individual semi-structured online interview if staff availability meant a focus group could not be scheduled. All patients who had been invited to the CR programme at participating sites (regardless of whether they actually ‘attended’) were invited to take part in a focus group or interview during the final two weeks of their CR programme. Potential participants were approached in person, by email or post by a member of the clinical CR team, and responded directly to JJ who provided the PIL and consent form. Potential participants completed a brief questionnaire to facilitate purposive sampling and ensure participant diversity of CR experience (e.g., mainly remote, mixed, mainly in-person), and sociodemographic characteristics (age, gender, ethnicity, socioeconomic status), although in practice (due to small numbers of patients expressing an interest in participating in the qualitative work), no sampling was undertaken and all interested patients were recruited. Seventy recruitment approaches were made to patients across the three sites.

### Data collection

Data collection took place between 13/12/2023 and 15/03/2024. Participants were not known to researchers before the study, and participants’ knowledge about the researchers related only to their involvement in the study. No interviewer or facilitator characteristics were reported to research participants. Topic guides were developed in advance and reviewed by the study Patient and Public Involvement (PPI) group before use. Topic guides were the same for focus groups and interviews but tailored to participant group: staff were asked about their experience of delivering hybrid CR and its perceived effectiveness; challenges encountered and recommendations for future implementation. Patients were asked about their experience of receiving CR; barriers and facilitators associated with following the hybrid CR pathway and perceived improvements that could be made to the Active^+^me REMOTE programme.

Two female health researchers with doctoral and post-doctoral experience of qualitative methods undertook data collection. For the focus groups, one researcher (JJ) facilitated sessions and the other (SD) made detailed notes. Interviews were conducted by JJ alone. All sessions were remote, and audio-recorded, with recordings transcribed verbatim by an independent transcriber. Only the participants and researcher(s) were present during data collection, and all participants took part in a focus group or interview on a single occasion.

### Data analysis

Data analysis was led by JJ with input from the wider research team. Data were managed using NVivo 14.0 (www.lumivero.com) and analysed thematically using a combination of deductive and inductive analysis. Deductive coding is appropriate when an existing theory or theoretical framework is used as the basis for data interpretation. In this case, due to our focus on understanding how hybrid CR could be implemented within health services, staff data were analysed deductively drawing on the broad domains of the Consolidated Framework for Implementation Research (CFIR) [20] (Table 2).

**Table 2:** Summary of CFIR domains.

Domain	Description
Innovation domain	The Active^+^me REMOTE app, including adaptability and usability
Outer setting domain	Relationships between the wider hospital system, app developer and CR team, including unanticipated events occurring between parties
Inner setting domain	Service organisation in the participating CR sites, including insights into perceived patient needs, and staff beliefs about hybrid CR
Individuals’ domain	Participants’ experiences of delivering hybrid CR and perceptions of patient experience and use of Active^+^me REMOTE
Implementation domain	Barriers and facilitators of hybrid CR

In contrast, patient data were analysed thematically, using inductive methods based on the approach described by Braun and Clarke [21] and without any preconceived codes or categories imposed on the data. New themes were developed iteratively and integrated as data collection progressed, with disagreements resolved through discussion. Study participants did not have the opportunity to review their transcripts or provide feedback on the results.

### Ethical approval and consent to participate

Ethical approval was obtained from the Wales 7 Research Ethics Committee (Ref: 23/WA/0131) on 9^th^ May 2023. Research governance approval was obtained from each participating site between 28/09/2023 and 12/01/2024. Formal, written consent was provided by all participants (electronically or by post) before their focus group or interview.

### Patient and public involvement

The National Institute for Health and Care Research (NIHR) Applied Research Collaboration (ARC) West Midlands PPI group contributed to study design and choice of methods. A Patient Advisory Group (PAG) comprising five members (some with lived experience of CHD) met regularly throughout the study. The PAG reviewed and commented on all study documentation and contributed to interpreting the qualitative data at a dedicated meeting convened after preliminary analysis.

## Results

A total of 12 staff across three sites (two focus group discussions, n = 10; two individual interviews) and six patients across three sites (all individual interviews) participated in the study. Focus groups lasted between 34 and 38 minutes; interviews lasted between 21 and 38 minutes. Staff participants were a mix of males (n = 6) and females (n = 6) and white (n = 9) and non-white (n = 3) ethnicities. Staff roles included nurse specialists (n = 3), cardiac physiotherapists (n = 2), heart failure rehabilitation nurses (n = 2), administrative staff (n = 3) and CR exercise instructors (n = 2). Patient participants were all male, aged between 40-59 (n = 4) or between 60-74 (n = 2). Five patients were of white ethnicity; one was of Asian heritage. Findings are presented thematically, with verbatim quotations.

### Findings from the staff interviews and focus groups

#### Innovation domain.

All staff reported initial concerns about how hybrid CR would integrate with their standard centre-based programme and the potential additional staff resource needed to implement hybrid CR. However, as familiarity with the Active+me REMOTE app and staff confidence increased, these concerns were allayed and hybrid CR was fully integrated at all three sites to form the standard CR offer. Staff participants all recognised that the app included more features than they were able to use during the initial implementation phase, and anticipated exploring additional features as hybrid CR became established as routine care:

*“There isn’t an awful lot in it for us at the moment, but I think that we probably don’t use it to its full capacity. I think there’s a lot more that we can do with it in the future.”* (Focus group, site 2)

At all sites, app training was delivered by the product developers to one or two staff, so that these staff could ‘onboard’ patients enrolling into the hybrid CR programme and cascade the training to other staff at their site. Nevertheless, staff at all sites described familiarising themselves with the app to understand its capabilities and explain it to patients:

*“I was set up with my own patient account on there within the training group and basically kind of experimented with the app.”* (Staff interview site 3)

#### Outer setting.

Implementing the externally-developed Active^+^me REMOTE app into NHS IT infrastructure was identified as a challenge at all sites. At one site, the research governance process was lengthy and required a dedicated project manager to facilitate approvals and set-up, which delayed the integration of hybrid CR into clinical practice:

*“All the governance issues around putting something in place, particularly that’s IT, particularly if you’ve got the information governance risks and all that goes with it. That was what probably really held things back.”* (Focus group, site 1)

#### Inner setting.

Staff all recognised that the ability for patients to have clinical readings measured in their home environment and uploaded for review by healthcare professionals was extremely beneficial. This was particularly the case for blood pressure data, where ‘white coat’ hypertension often means that readings taken at in-person CR clinics are inaccurate. Patients could access or upload information at any time, which was welcomed by staff in reducing time and staff pressures associated with seeing patients in person:

*“It’s definitely the time for us to be going more digital and not seeing patients in clinic often… working remotely and via the app is really good.”* (Focus group, site 1)

However, the app function which allowed patients to participate in education sessions from home synchronously with ‘live’ centre-based sessions was less effective given that participating in live sessions was limited to patients who were available to login at a specific date and time, which often resulted in poor attendance. Having education sessions available asynchronously was an app function that had not been exploited fully by participating sites but was seen as potentially beneficial to patients:

*“Our education is virtually every six weeks on Microsoft Teams and usually we have 15, 20 patients signed up but, on the day only about five turn up, so put that on the app.”* (Focus group, site 2)

#### Individuals’ domain.

Two sites initially offered Active^+^me REMOTE as a standalone pathway that patients could select as an alternative to the traditional, centre-based programme. However, these sites reported that uptake of hybrid CR offered in this manner was extremely low, prompting them to offer Active^+^me REMOTE routinely to all patients as an addition to the standard pathway:

*“99% of our patients are using it in combination with something else. So, they are not using just the Active*^*+*^*me app, they’re coming to classes and then using the Active*^*+*^*me to monitor things at home”* (Interview, site 3)

Staff at all sites were initially selective about the patients they invited to participate in hybrid CR. Patients with poor internet access were considered unable to participate, and staff felt a clinical responsibility to offer hybrid CR only to those patients they felt could cope with it physically. This meant that some older patients, those with mental health issues or poor literacy skills (including perceived digital literacy) may have been steered towards centre-based CR by staff. However, as staff confidence in delivering hybrid CR increased over time, staff reported feeling less pressure to screen patients before enrolment:

*“When we first started getting people involved with the app, we were more or less cherry-picking and the people that we were […] interested in were people that were IT-savvy and they were fairly young fellas mainly and they were the ones, they were our guinea-pigs. But as time has gone on, I feel a lot more confident about uploading or getting people involved that are not the sort of fitter ones; they’re the ones that are needing a little bit more in terms of monitoring.”* (Focus group, site 2)

Some staff avoided offering Active^+^me REMOTE to patients they perceived as being ‘obsessive’ about their health, concerned that the wealth of data available through the app could heighten patient anxieties about their progress:

*“I’m looking for people that have got online access because that is a big thing, but I try to avoid the obsessive types, because they’re already obsessing about things and you don’t want to intensify that.”* (Focus group, site 2)

All staff agreed that a key benefit of hybrid CR is its role in helping patients to better understand their condition and encouraging them to manage their own health and recovery:

“*Excellent concept - we want patients to be self-caring and self-monitoring. Giving them responsibility of their own health instead of it coming down to the nurse or the doctor.”* (Focus group, site 1)

However, staff reported varying levels of engagement with the app amongst patients that had registered for it. Consequently, there was some concern over the long-term engagement of patients with the app and hybrid CR in general:

*“But […] the long-term engagement, life takes over some people and I think it’s mainly for the younger people who are going back to work, they haven’t got time to have a look, you know, but I think our older generation seem to have took it on board really well.”* (Focus group, site 1)

#### Implementation process domain.

An app security feature automatically logged users out after a short period of inactivity, which was reported as frustrating and time-consuming by staff attempting to login and review patient data. The app was also slow in retrieving patient data which became more problematic as the number of enrolled patients increased:

*“I think the main difficulty has been the delay and I don’t know whether it’s a firewall issue but in terms of getting on the app, it locking you out, accessing records, that has been time consuming. It takes such a long time for you to access it, to get the data to come up, it’s very slow when we’re working at a fast pace all the time.”* (Focus group, site 1)

However, the app developers were responsive to these concerns and developed a new ‘dashboard’ view so that staff could see all relevant information about a patient in one place, which staff found helpful:

*“So, they did that in response to us saying how time consuming it was to find all the bits of information needed on that one patient, whereas now you can just see it at a glance.”* (Focus group, site 1)

Staff at site 1 also reported that some patients had issues with the wearable devices that had been issued (blood pressure cuffs in particular). These were often faulty and needed to be replaced:

*“They’re not cheap blood pressure cuffs to have so many errors, given that we don’t send everybody out with one because a lot of people have got their own, but out of the ones that have had them, it’s been notable the amount of people that have said they’ve got problems with them.”* (Focus group, site 1)

### Findings from the patient interviews

Thematic analysis resulted in two main themes, each with two sub-themes. The first theme concerned patients’ engagement with hybrid CR (including: i) patient views about app usability, and ii) broader views about hybrid CR). The second theme focused on patients’ holistic health (including: i) psychological wellbeing, and ii) future health).

#### Engagement with hybrid CR.

*Patient views about the usability of the Active*^*+*^*me REMOTE app:* Most patients felt that the Active^+^me REMOTE app was reasonably easy to use and valued the automatic transfer of data from wearable technology (e.g., blood pressure monitors) to the app. Patients felt reassured that CR staff were monitoring clinical data and that any anomalies would be identified promptly. Patients who had resumed full-time employment found app access extremely important for their continued rehabilitation:

*“I work a long day... so it would be like come to work at seven and by the time it would be seven (pm) I thought no, you know, but having that app, I know it sounds really stupid, but it just made me feel, I don’t know just made me feel I’m still being monitored”* (Patient interview site 2)

However, a small number of patients identified issues with the functionality of the app in terms of being able to see their exercise progress over time and not knowing what was happening to the monitoring data that was being uploaded via the wearable devices such as blood pressure monitors:

*“Probably my main criticism with it really is that that bit did feel really clunky. And then when you actually wanted to review your exercising and see what was recorded you couldn’t actually really see, or I haven’t been able to really see what I did on what day to be able to compare one day’s exercise against another day’s exercise. I wouldn’t say I was fed up with it, but sort of it was just recorded and you’re sort of thinking well where’s this data going? Who’s seeing it?”* (Patient interview site 3)

A small number of patients also raised issues about the need for safety questions to be answered whenever they were embarking on self-directed exercise. These questions could not be skipped and were seen by some patients as unnecessary and repetitive when the same checklist needed to be completed before every exercise:

*“I don’t think this is data you need to record. When I’m doing a walk I find the list of questions “Yes I’ve got medicine. Yes I’ve done a warmup. Yes I’ve done that”. Just let me get, just let me say yes. I read these every day. And I’ve kind of stopped paying attention to them, just tick all the boxes.”* (Patient interview, site 1)

*Patients’ views of hybrid CR:* Whilst acknowledging that hybrid CR was empowering to patients, participants also noted the possibility of becoming ‘obsessed’ with monitoring their clinical data:

“*If you get sort of OCD about it really…I’ve lost probably half a stone and then you put two pounds back on and you go ‘oh my goodness, that wasn’t supposed to happen!’”* (Patient interview site 2)

Overall, participants felt that being able to combine in-person contact and the app worked well. Although in-person sessions remained the preferred option for some patients, the app gave opportunities to maintain recovery without needing to attend group sessions, as long as individuals had enough motivation to use the app to its full potential:

*“I was doing it online when it was convenient for me. So that’s very important, I think. If you are committed and motivated, then you can really capitalise on that.”* (Patient interview, site 1)

#### Patients’ holistic health.

*Psychological wellbeing:* Patients felt that in-person sessions provided good advice, were well organised, and catered for different abilities within each group. Patients valued the social aspect of these sessions and reported how helpful it was to meet others with similar experiences:

*“I’ve thoroughly enjoyed the classes and the meetings and the rehab in the group sessions. The app – probably haven’t enjoyed that so much……You’re given it and said this is what you’ve got, and you can use it and learn it yourself.”* (Patient interview, site 2)

One patient had experienced both centre-based (from a previous cardiac event) and hybrid CR and had found the hybrid pathway to be more informative than their previous experience. However, some patients were anxious about having suffered a cardiac event and felt they would have benefited if the hybrid programme included better psychological support:

*“I think with a heart condition as well I think you panic that oh my god I’m going to die at any minute, I can’t do this, I can’t do that, I can’t, you know, and I think you go into a freefall of emotion and I think if people were talked through what they could – the steps that you were going through – almost like a denial, anger, those sort of things.”* (Patient interview, site 2)

*Future health:* All patients described how hybrid CR had given them valuable insight into their health and treatment. For some, it was useful to understand the correct way to exercise to improve fitness by maintaining their heart rate at the optimal level within the parameters set by the CR team.

*“I honestly thought that you know, going hell for leather improved your fitness but it just goes to show.”* (Patient interview, site 2)

Using the app encouraged patients to take ownership of monitoring their health, with a number of patients using their app data as a record to demonstrate progress to other healthcare professionals outside of the CR team:

*“So, the app gives you a recording of blood pressure. Now the local health centre don’t see that data but it enabled me to show them; ‘here’s the readings I’ve done over the last three days.’”* (Patient interview, site 3)

### Synthesis of staff and patient findings

Table 3 summarises the barriers and facilitators to hybrid CR as identified by staff and patients.

**Table 3:** Barriers and facilitators to hybrid CR (staff and patients).

Item	Patients	Staff
**BARRIERS**		
Digital literacy	No	Yes
Internet access	No	Yes
Difficulties using app on smartphone for online classes	Yes	Yes
Social isolation when using app	Yes	Yes
Login issues	Yes	Yes
Safety questions asked before exercise sessions	Yes	No
App unable to retain detailed activity records	Yes	Yes
**FACILITATORS**		
Ease of use	Yes	Yes
Increased patient knowledge about their condition	Yes	Yes
Empowering patients to take control of their health	Yes	Yes
Prompt and effective support from app developers	Yes	Yes
Flexibility for patients in how and when they work through rehabilitation	Yes	Yes
Opportunity to engage with CR after returning to work	Yes	Yes

## Discussion

The introduction of hybrid CR, in which in-person CR sessions are combined with remote delivery, has the potential to improve CR uptake and adherence in comparison to centre-based CR [22–25], although there is limited evidence about the degree to which attrition rates from patients in groups that may be under-represented in CR services are similar in hybrid programmes to those seen in centre-based programmes [26]. Emerging evidence suggests that clinical outcomes from hybrid CR are comparable to centre-based CR [23,27–29], although cost-effectiveness is less clear [23,30].

Several qualitative studies of hybrid CR have been undertaken, although ours is among the first to assess hybrid CR outside of the COVID-19 pandemic and across multiple sites. Our staff data demonstrated positive engagement with hybrid CR and a belief that a flexible, digitally-enhanced CR offer was effective despite some technical issues described by a small number of participants. Other studies have found that an important facilitator for hybrid CR is a strong relationship between patients and staff [31], with effective hybrid CR dependent on CR team culture and staff desire to ‘champion’ the hybrid approach for patients [32]. Our staff participants expressed some concerns about workloads associated with offering both centre-based and app-based CR. The introduction of technology into centre-based CR services and its impact on staff resources has also been recognised by others, particularly during early implementation phases [33,34]. Sufficient time must be allowed for system learning, staff training and the creation of new clinical practice routines when introducing hybrid pathways to ensure that system pressures do not inhibit adoption [33,34].

The literature on digital CR shows that digital literacy and technology confidence are important predictors of patient engagement [35]. Our findings suggest that hybrid CR was acceptable and could be tailored by patients to meet their needs and personal circumstances. The opportunity to provide personalised programmes for patients based on individual risk factor assessment is a key strength identified by our work and in the wider hybrid CR literature [36,37]. Our findings did not suggest that digital literacy was a particular concern to patients. In contrast, digital literacy was highlighted by some staff as guiding decisions that they made about which patients should be offered the opportunity to follow the hybrid CR pathway. Similarly, although internet access was not a concern to patients, staff were unlikely to offer hybrid CR to any patients that they felt did not have sufficient internet access to use the app effectively. Given that hybrid CR is typically considered a way to overcome some of the inequities in access to CR that are seen in conventional rehabilitation programmes, it is important that hybrid CR does not introduce additional inequalities in patient access to services. Both patients and staff reported technical issues with logins and app functionality, as has been noted by others [22,38,39]. It is important that such issues are addressed effectively so that patients do not become disillusioned with remote technologies and disengage from CR as a result.

The effectiveness of technology-enabled CR for patients is closely associated with perceived usability, usefulness and acceptability of the technology and how this interfaces with in-person CR delivery [31]. Despite a small number of issues reported relating to app logins and some frustration with the need to respond to a checklist of safety questions before commencing an exercise session, our patient data showed that the Active^+^me REMOTE app was easy to use, convenient, and that activity tracking functions were highly valued. This aligns with other work that highlights the sense of control and empowerment patients can gain from participating in technology-enabled CR programmes [38,40], and suggests that hybrid CR can accommodate diverse patient preferences and expectations. Patients have also reported that the self-monitoring capabilities offered by technology can give reassurance about their health status [38]. However, limited opportunities for social interaction may raise challenges for patients seeking peer support whilst participating in CR if this is not available as a remote function [33]. Staff recognised that they had not used the peer support function in Active^+^me REMOTE and acknowledged its potential benefit. There is also a danger that having access to large amounts of monitoring data may be problematic for those with a tendency to ‘obsess’ over their progress.

The barriers to hybrid CR may differ from those associated with centre-based CR [41], and it is important that hybrid pathways do not exacerbate inequalities and worsen the under-representation of some groups within services [42]. Hybrid CR can overcome challenges relating to transport, geography and time, but challenges to engagement may remain for those with poor technology access, poor digital literacy or low technology confidence [43]. Indeed, some staff noted feeling a responsibility to ‘screen’ patients and offer the app only to patients they felt could cope with technology-enabled CR, although such screening lessened as staff confidence in using the app increased. This raises a concern about the degree to which hybrid CR is able to address potential equity concerns in access to rehabilitation services and would need to be considered carefully by services implementing a fully hybrid approach. The sustainability of hybrid services is also important. Whilst our patient participants all wanted to continue their recovery using the app, the small number of staff trained on Active^+^me REMOTE at each site made the ongoing offer of hybrid CR vulnerable to issues such as staff turnover or sickness. This study considered the implementation of hybrid CR at three sites and as such could not assess issues around scalability of hybrid CR approaches. However, it is clear that the costs and resource implications of hybrid CR would need to be assessed in detail, alongside the capacity of the app developers to respond in a timely manner to technical issues. Issuing wearable technology to patients following a hybrid CR pathway would also need to be considered from a cost perspective.

### Limitations

Whilst all study sites offered hybrid CR using Active^+^me REMOTE as standard, each site engaged with the app’s capabilities differently, with some using limited functions or using hybrid delivery only for specific CR components, which makes comparison across sites challenging. With some sites imposing baseline requirements for digital literacy that impacted on which patients were offered hybrid CR, this may limit the generalisability of our findings and have implications for the extent to which the hybrid CR offer can be scaled up and adopted more widely without compounding existing inequities in access to services. Our number of patient participants was small, and the sample may not have been representative of the wider population undertaking CR at participating sites (interviewees were affluent, all were male and 5/6 were of white ethnicity). The influence of factors such as socioeconomic status, sex and ethnicity may have fundamental impacts on patient views and experiences of hybrid CR and service equity, and our limited sample impacts the generalisability of our results. However, all sites were represented in our data and we reached thematic saturation in both participant groups. We could not interview any patients who chose not to participate in hybrid CR or who dropped out after enrolment. This limitation is frequently reported in similar studies, although we did gain a perspective on patient non-participation and non-adherence from our staff participants.

## Conclusions

The Active+me REMOTE app for CR was acceptable, convenient and flexible in its functionality, allowing support to be tailored to the needs and capabilities of individual patients and patient progress to be monitored by staff. Findings imply that the effective implementation of hybrid CR requires resources at the system level to allow hybrid pathways to be embedded as routine care so that system pressures do not inhibit adoption. It is also important that sufficient time is allowed for staff training to maximise staff members’ confidence and ability to enrol patients to receive hybrid CR as the standard service offer rather than screening patients to follow alternative CR pathways. To address equity concerns, services might consider a structured assessment of digital access and digital literacy. Staff need allocated time to contact patients who cease activity on dashboards to identify adherence issues and offer in-person CR if barriers to the remote components are identified.

## Supporting information

S1 Table COREQ (COnsolidated criteria for REporting Qualitative research) checklist. (DOCX)
